# Alternative Substrate Metabolism in *Yarrowia lipolytica*

**DOI:** 10.3389/fmicb.2018.01077

**Published:** 2018-05-25

**Authors:** Michael Spagnuolo, Murtaza Shabbir Hussain, Lauren Gambill, Mark Blenner

**Affiliations:** ^1^Department of Chemical and Biomolecular Engineering, Clemson University, Clemson, SC, United States; ^2^Program in Systems, Synthetic, and Physical Biology, Rice University, Houston, TX, United States

**Keywords:** *Yarrowia lipolytica*, pentose, hexose, xylose, fat, waste, acetate, metabolic engineering

## Abstract

Recent advances in genetic engineering capabilities have enabled the development of oleochemical producing strains of *Yarrowia lipolytica*. Much of the metabolic engineering effort has focused on pathway engineering of the product using glucose as the feedstock; however, alternative substrates, including various other hexose and pentose sugars, glycerol, lipids, acetate, and less-refined carbon feedstocks, have not received the same attention. In this review, we discuss recent work leading to better utilization of alternative substrates. This review aims to provide a comprehensive understanding of the current state of knowledge for alternative substrate utilization, suggest potential pathways identified through homology in the absence of prior characterization, discuss recent work that either identifies, endogenous or cryptic metabolism, and describe metabolic engineering to improve alternative substrate utilization. Finally, we describe the critical questions and challenges that remain for engineering *Y. lipolytica* for better alternative substrate utilization.

## Introduction

The oleaginous yeast *Yarrowia lipolytica* has great potential for the production of a large range of biochemicals and intermediates. As an oleaginous yeast, its metabolism is well-primed for the biosynthesis of triacylglycerides (TAGs) when grown in nutrient-limited conditions. This requires high flux through acetyl-CoA, making it a good production host for molecules that likewise require acetyl-CoA. There are numerous examples of metabolic engineering efforts to make economically useful amounts of products, including TAGs, omega-3 rich TAGs, fatty acid derivatives, carotenoids, and organic acids (Yuzbashev et al., 2010; Xue et al., 2013; Blazeck et al., 2014; Qiao et al., 2015; Ryu et al., 2015; Wang et al., 2016; Xu et al., 2016; Gao et al., 2017; Kildegaard et al., 2017; Sabra et al., 2017; Schwartz et al., 2017a; Xie et al., 2017; Sagnak et al., 2018). Several recent reviews have thoroughly covered metabolic engineering efforts, as well as advances in the genetic engineering toolkit that enables such work (Blazeck and Alper, 2013; Hussain et al., 2016; Adrio, 2017; Yan et al., 2017). In most cases, refined glucose is used as the substrate for *Y. lipolytica* growth and product formation. There are many alternative substrates that can and should be considered due to their relative low costs, additional sustainability benefits, and in some cases, better conversion to products. The use of polysaccharides, less-common monosaccharides, and other select substrates, as well as the associated engineering efforts, have been covered in a recent review (Ledesma-Amaro and Nicaud, 2016a). In this review, we expand on the set of alternative substrates that have been used for *Y. lipolytica* growth and bioproduction. We further provide examples where metabolic engineering and synthetic biology were applied to enhance alternative substrate utilization.

## Hexose Substrates

### Mannose

Mannose is a C-2 epimer of glucose and belongs to the family of aldo-hexoses. In nature, it exists in either its predominant, sweet tasting α-form or in its less common, bitter tasting, β-anomeric form. D-Mannose is commonly used as a nutritional supplement and, in some instances, as an anti-fungal therapy because of its relative toxicity. Mannose has been shown to be readily fermented by *Saccharomyces cerevisiae* (Gottschalk, 1946; Du Preez et al., 1986), although it has been the subject of relatively fewer studies in comparison to other aldo-hexose sugars. While metabolism of mannose is known to occur in *Y. lipolytica* (Coelho et al., 2010), the complete metabolic pathway has not yet been fully elucidated.

**FIGURE 1:**
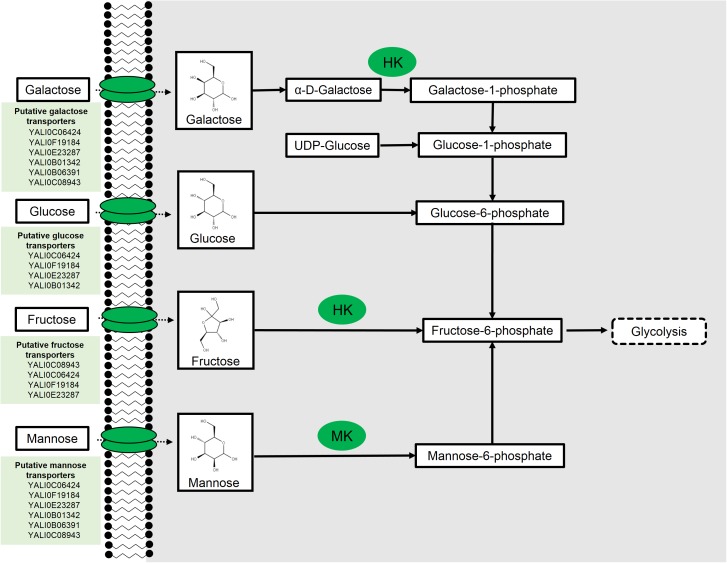
Hexose metabolism in *Yarrowia lipolytica*. Hexokinase (HK) and mannokinase (MK). Putative transporter results from Lazar et al. (2017).

Following transport of mannose into the cell, one pathway for mannose utilization is by isomerization to fructose; however, such isomerases are predominantly found in bacteria and do not exist in yeast (Rajesh et al., 2012; Sigdel et al., 2015). Not surprisingly, BLAST analysis revealed no homologs of bacterial mannose isomerases in *Y. lipolytica* (all BLAST analysis performed under default parameters unless source is noted). A more common mechanism for mannose metabolism is specific mannose phosphorylation to mannose-6-phosphate by a mannokinase (MK2, **Figure 1**). These enzymes are found in some bacteria and higher-level eukaryotic systems (Fukuda et al., 1984). BLAST analysis again revealed no homologs in *Y. lipolytica*, suggesting the absence of a specific enzyme. In *S. cerevisiae*, mannose is phosphorylated by hexokinases (HKs), which have broad specificity. Two of these HK isozymes, HXK1 and HXK2, act on a broad range of sugars including keto and aldohexoses (Rodriguez et al., 2001; Subtil and Boles, 2012). Meanwhile the third isozyme, glucokinase (GLK1), possesses a more stringent specificity toward aldohexoses, including mannose, and lacks activity toward fructose (Maitra, 1970; Gancedo et al., 1977). BLAST analysis of the *S. cerevisiae* glucokinase and HK revealed two orthologs in *Y. lipolytica*, *ylHXK1* (YALI0B22308g), and *ylGLK1* (YALI0E15488g). Given the promiscuity of the kinases in *S. cerevisiae* (Sharifpoor et al., 2011), it is likely that these two HKs are involved in mannose phosphorylation, although this has yet to be directly shown.

While the use of mannose as a carbon source has received limited attention, the role of mannose in glycoprotein biogenesis has been well studied (Ezekowitz et al., 1990; Chiba et al., 1998; Hamilton et al., 2003; Zhu et al., 2009; Tiels et al., 2012). More recently, *Y. lipolytica* has been explored as a host for the production of therapeutic mannoproteins due to its efficient heterologous protein secretory capabilities and the growing set of genetic tools available, such as a suite of promoters and terminators (Thevenieau et al., 2009; Blazeck et al., 2011, 2013; Curran et al., 2015; Shabbir Hussain et al., 2016, 2017), pooled promoters (Dulermo et al., 2017), secretion markers (Nicaud et al., 2002; Madzak et al., 2004), Golden Gate assembly (Celińska et al., 2017), and CRISPR systems (Gao S. et al., 2016; Schwartz et al., 2016, 2017b). Mannosylphosphorylated *N*-glycans are a precursor to produce mannose-6-phosphate containing glycoproteins. A single gene in *Y. lipolytica*, *ylMpo1*, was found to efficiently catalyze a mannophosphorylation reaction in a single step (Park et al., 2011) whereas this reaction is a two-step enzymatic process in *S. cerevisiae* (Gil et al., 2015). While *N*-glycosylated mannoproteins are outside the scope of this review, the above example demonstrates one method by which *Y. lipolytica* could effectively assimilate mannose into protein therapeutics.

### Galactose

Galactose is a C-2 epimer of glucose and belongs to the family of aldo-hexoses. D-Galactose is the only natural isomer and is abundant in plant cell biomass and milk. The most predominant mechanism for galactose metabolism in yeast is the Leloir pathway. This pathway shuttles galactose through a series of sequential enzymatic steps to produce glucose-6-phosphate that can then be funneled into glycolysis. *S. cerevisiae* has been used as a model organism for complete characterization of the Leloir pathway (Douglas and Hawthorne, 1964; Ostergaard et al., 2000; Timson, 2007). Another pathway for galactose metabolism has been identified in filamentous fungus, *Aspergillus niger*, that involves a series of energy intensive redox reactions to convert galactose to D-fructose (Mojzita et al., 2012).

Recently, all enzymes implicated in the Leloir pathway were identified in the *Y. lipolytica* genome; however, the French strain, W29 and the corresponding PO1 auxotrophs, do not grow on galactose substrate despite the presence of predicted Leloir pathway genes (Lazar et al., 2015). Lazar et al. (2015) determined that overexpression of three native genes, *ylGAL1* (*YALI0C13090g*), *ylGAL7* (*YALI0F23947g*), and *ylGAL10E* (*YALI0E26829g*), were needed for growth on galactose (**Figure 1**). Expression of these genes under the strong, constitutive translation elongation factor 1-α (TEF1-α) promoter resulted in growth comparable to growth using glucose. Furthermore, growth on glucose and galactose enabled galactose uptake, although glucose was metabolized at a relative higher rate. Complementation of the three *ylGAL* genes in the Δ*scGAL* strain restored galactose metabolism confirming that the identified *Y. lipolytica* orthologs are, in fact, genes belonging to the Leloir pathway.

While the structural components of the Leloir pathway exist in *Y. lipolytica*, a BLAST analysis for *S. cerevisiae* orthologs of regulatory proteins, namely *scGAL3*, *scGAL4*, *scGAL80*, and *scGAL11*, were not identified (Suzuki et al., 1988; Lazar et al., 2015). Furthermore, the 17 bp *scGAL4* consensus binding sequence was also not found in any of the *ylGAL* promoters. These results suggest that promoters regulating the *Y. lipolytica* genes in the Leloir pathway are not inducible by galactose in contrast to *S. cerevisiae* Leloir pathway genes (John and Davis, 1979; Schultz et al., 1987). A plausible explanation for the above results is that there is a cryptic regulatory mechanism in *Y. lipolytica* that is evolutionarily divergent from *S. cerevisiae*.

Catabolite repression of galactose by glucose in *S. cerevisiae* has been extensively studied (Zimmermann and Scheel, 1977; Ma and Botstein, 1986; Escalante-Chong et al., 2015). This happens by repression of the *GAL3* transcriptional factor necessary for inducing the *GAL* gene cluster (Bajwa et al., 1988). scGAL3 is an ohnolog of scGAL1 that has evolved to function as a transcription factor by loss of two amino acids that abrogate its enzymatic activity (Platt et al., 2000). Lazar et al. (2015) demonstrated that ylGAL1 is not a transcriptional regulator, but rather only possessed HK activity. The fact that all identified *ylGAL* genes were expressed in glucose strongly suggests that *Y. lipolytica* regulates galactose with a very different mechanism compared to *S. cerevisiae* or other fungal species such as *A. nidulans* (Drysdale et al., 1993).

### Fructose

Fructose is a ketohexose found predominantly in sweet fruits and honey that has been used as a sweetener, as well as a preservative. Fructose metabolism is well understood, requiring phosphorylation of fructose to fructose-6-phosphate by a HK (**Figure 1**). In *Y. lipolytica*, ylHK1 is mainly responsible for catalyzing this step. Overexpression of *ylHK1* resulted in up to 55% lipid accumulation from fructose under nitrogen-limited conditions (Lazar et al., 2014).

Metabolism of fructose in *Y. lipolytica* has gained much attention because of its widespread abundance and low cost in molasses, sucrose, and corn syrup. The disaccharide sucrose is made of the monosaccharides glucose and fructose. *Y. lipolytica* is naturally unable to metabolize sucrose because it lacks an invertase enzyme required for the cleavage of the disaccharide. The recombinant *scSUC2* gene was first used as a positive selection marker for screening of *Y. lipolytica* transformants (Nicaud et al., 1989a) and more recently, was integrated into common W29-derived strains, such as Po1f (Nicaud et al., 1989b). *scSUC2* expression and secretion was enhanced using the strong *pTEF* promoter to drive expression and an optimized signaling sequence (Hong et al., 2012). This new *Y. lipolytica* strain resulted in superior intracellular and extracellular invertase activity followed by more robust growth on sucrose (Lazar et al., 2013).

With the extracellular secretion of invertase, *Y. lipolytica* grown on sucrose was able to uptake both glucose and fructose, although glucose was preferred (Moeller et al., 2012). When *Y. lipolytica* was grown on either fructose or glucose as the sole carbon source, however, uptake of both carbon sources occurred at the same rate, hinting that yeast possess similar uptake and transport mechanisms for glucose and fructose. Furthermore, Moeller et al. (2012) showed that the glucose uptake rate was reduced when fructose was already present in the culture media. Fructose concentrations ranging from as low as 5 g/L to as high as 85 g/L were tested and resulted in glucose uptake inhibition.

### Hexose Transporters

Because sugars are not able to freely transport across the cell membrane, an active transport mechanism is needed to facilitate the process. Transporter proteins exist at the interface of the cell membrane that allow for the selective uptake of sugars. In yeast, these transporters tend to be more promiscuous, where generally one transporter is capable of transporting multiple types of sugar often with preference for one (Colabardini et al., 2014; Jordan et al., 2016).

In *Y. lipolytica*, there are at least 24 sugar transporters with 6 of these being identified strictly as hexose transporters (Lazar et al., 2017). These hexose transporters were identified from heterologous expression of the *Y. lipolytica* transporters in a knockout strain of *S. cerevisiae* incapable of sugar uptake. These transporter findings are summarized in **Figure 1**. While such experiments are common, they can miss true positives due to either poor expression or folding in heterologous systems, leading to a measured lack of functionality. Another interesting fact revealed by Lazar and team was the existence of inter-strain variability toward the uptake of fructose between the W29 and the H222 German strain. The W29 strain was less capable of growth in fructose compared to H222, which demonstrated robust growth in fructose as the sole carbon source (Lazar et al., 2014). Mining of the draft genome of both W29 and H222 revealed a pseudogene (*YALI0C04686g*) in W29 with strong sequence similarity to H222’s functional fructose transporter. Furthermore, amino acid polymorphisms were found in eight of the transporters between the two strains that could potentially explain an evolutionary divergence of the H222 strain that resulted in modifications to transporters that enable better fructose uptake.

Sugar transporters have been extensively studied in *S. cerevisiae* (Luyten et al., 2002; Batista et al., 2004; Rintala et al., 2008). There are 20 identified genes in the family of hexose transporters for *S. cerevisiae*, of which 18 are bona fide transporters (*HXT1-17* and *GAL2*) and two genes encode sensor proteins (*SNF3* and *RGT2*). The expression pattern of these genes is also strongly dependent on oxygen conditions (Rintala et al., 2008). Furthermore, the regulation and expression of these transporters are highly complex, requiring the involvement of several transcription factors. One strategy to improve glucose uptake rate, and thereby improve biochemical production, is to overexpress the high affinity *HXT7* transporter (Kim et al., 2015). BLAST analysis of the *scHXT7* protein against the *Y. lipolytica* genome revealed three of the six currently identified hexose transporters (YALI0F19184p, YALI062424p, and YALI0C09843p) with the former having highest sequence homology to *HXT7*. The same group showed that the rate of glucose uptake was improved with the overexpression of the GCR1 transcription factor due to its role in inducing the low-affinity HXT1 transporter thought to be critically important at the beginning of fermentation (Luyten et al., 2002). BLAST analysis for a homolog of GCR1 in *Y. lipolytica* yielded no matches, indicating a potentially divergent mechanism for regulation. Finally, SNF1 is a sensor required for high-affinity glucose transport into the cell for *S. cerevisiae* (Bisson, 1988). A BLAST search for SNF1 homologs in *Y. lipolytica* revealed the three above-mentioned genes being the top candidates. This further supports the theory that the mechanism for regulation is more divergent from those seen in *S. cerevisiae* and it strengthens the case for the three aforementioned genes potentially being implicated in high-affinity glucose uptake in *Y. lipolytica*.

### Outlook

While sugar assimilation has been well-studied in the conventional *S. cerevisiae*, the uptake mechanisms, metabolic pathways, and regulatory mechanisms remain poorly understood in *Y. lipolytica*. To date, much of the work in *Y. lipolytica* has focused on identifying a few key players involved in hexose assimilation, while engineering toward improved uptake and metabolism of hexose sugars have not been explored as thoroughly. Additionally, efforts have been made to enable the consumption of polysaccharides such as starch (Ledesma-Amaro et al., 2015), cellulose (Wei et al., 2014), cellobiose (Guo et al., 2015), and hemicellulose (Yang et al., 2009). This current review attempts to draw parallels to *S. cerevisiae*, when possible, with the intention of elucidating potential pathways that could be explored moving forward. Understanding more about the regulatory pathways for hexose metabolism could enable engineering strains capable of improved hexose utilization.

## Pentose Substrates

### D-Xylose

**FIGURE 2:**
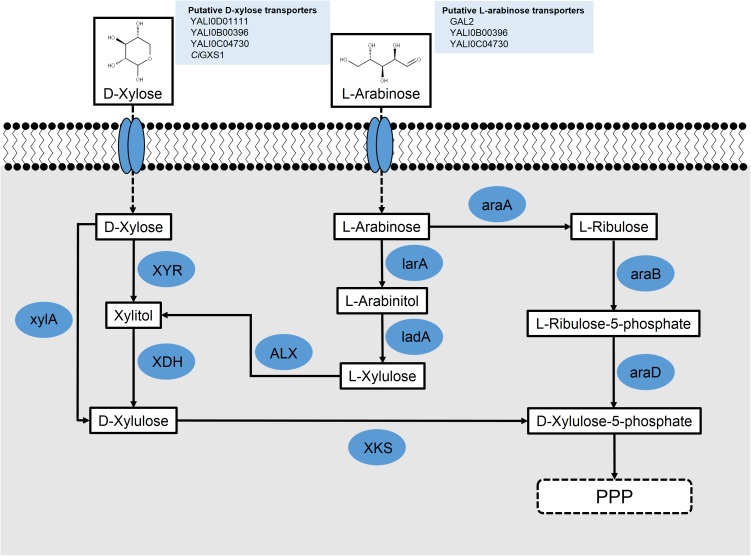
Schematic for the putative metabolism of pentose sugars L-arabinose and D-xylose in engineered *Y. lipolytica*; L-arabinose reductase (larA) (*Aspergillus niger*); L-arabinose 4-dehydrogenase (ladA) (*A. niger*); L-arabinose isomerase (araA) (*Bacillus subtilis*); L-ribulokinase (araB) (*Escherichia coli*); L-ribulose-5-phosphate 4-epimerase (araD) (*E. coli*); L-xylulose reductase (ALX) (*Ambrosiozyma monospora*); xylose reductase (XYR); xylose dehydrogenase (XDH); xylulokinase (XKS); and xylose isomerase (xylA).

As the most prevalent pentose sugar in hemicellulose, D-xylose is of great interest to the future of biofuels and a biomass economy. Many biotechnologically relevant microbes that can utilize glucose for growth and synthesis of value-added products cannot inherently metabolize D-xylose. With D-xylose making up a large portion of the sugars present in lignocellulosic biomass, enabling its metabolism in model microbial systems could significantly increase the economics of biomass utilization. D-Xylose is poorly metabolized by *S. cerevisiae*, but is efficiently utilized by *Scheffersomyces stipitis*, which serves as a model for xylose metabolism. Its metabolism in yeast commonly proceeds through the so-called oxidoreductase pathway; however, some anaerobic fungi have acquired the isomerase pathway through horizontal gene transfer from bacteria (Kuyper et al., 2003). These pathways are shown in **Figure 2**.

Significant effort has been spent to engineer xylose metabolism in *S. cerevisiae*. Heterologous expression of *S. stipitis* genes for xylose reductase (*ssXYR*) and a xylitol dehydrogenase (*ssXDH*) (Kotter et al., 1990) led to improved xylose metabolism. Further analysis of this engineered strain suggested that a third enzyme, a xylulokinase (*XKS*), is needed to improve xylose utilization and ethanol production (Toivari et al., 2001). Nevertheless, xylose metabolism through the oxidoreductase pathway remains challenging due to redox imbalance (Jin et al., 2004). A comprehensive review of engineered xylose metabolism in *S. cerevisiae* is available (Kim et al., 2013).

Recently, natural and engineered xylose metabolism has been studied in *Y. lipolytica*, with the help of genetic engineering and bioinformatics approaches. There have been inconsistent reports describing whether or not xylose could naturally be metabolized by *Y. lipolytica*. While some studies have described native xylose metabolism by *Y. lipolytica*, a majority of the accounts suggest that *Y. lipolytica* cannot utilize xylose without subjecting it to adaptation or starvation periods (Ruiz-Herrera and Sentandreu, 2002; Tsigie et al., 2011; Tai, 2012; Blazeck et al., 2014). Interestingly, these reports conflicted even among identical strains. In an effort to elucidate and induce xylose metabolism in *Y. lipolytica*, several genetic engineering approaches have been explored. The following four reports were published at approximately the same time. Results from these and other related studies are shown in **Table 1**.

**Table 1:** Summary of recent studies on native and engineered xylose metabolism in *Yarrowia lipolytica*.

	ssXYR	ssXDH	ssXKS	XYLA	XYR	XDH	XKS	Growth on xylose without adaptation?
					YALI0D07634	YALI0E12463	YALI0F10923	
Ledesma-Amaro et al., 2016b	✓	–	–	–	–	–	–	No
	–	✓	–	–	–	–	–	No
	✓	✓	–	–	–	–	–	No
	–	–	–	–	–	–	✓	No
	✓	–	–	–	–	–	✓	No
	–	✓	–	–	–	–	✓	No
	✓	✓	–	–	–	–	✓	Yes
Li and Alper, 2016	✓	✓	–	–	–	–	–	No
Li and Alper, 2016	–	–	–	✓	–	–	–	No
Rodriguez et al., 2016	–	–	–	–	–	✓	✓	Yes
Ryu et al., 2016b	–	–	–	–	–	✓	–	No
Tai, 2012; Stephanopoulos and Tai, 2015	✓	✓	–	–	–	–	–	No
Niehus et al., 2018	–	–	–	–	✓	✓	✓	Yes


Genome mining suggested that a complete xylose pathway was present in *Y. lipolytica*; however, growth on xylose was not observed in three studies (Ledesma-Amaro and Nicaud, 2016b; Rodriguez et al., 2016), and extremely weak growth was observed in a fourth study after a long period of adaptation (Ryu et al., 2016b). In these weakly growing strains, mRNA expression levels measured with quantitative polymerase chain reaction (qPCR) indicated inducibility of *ylXDH* gene (*YALI0E12463*), but overall weak expression (Ryu et al., 2016b). This observation led to their hypothesis that the *ylXDH* expression was limiting. Overexpression of *ylXDH* resulted in a significant improvement in growth on xylose after adaptation (Ryu et al., 2016b).

To better understand the intrinsic capability of *Y. lipolytica* for xylose metabolism, endogenous *ylXDH* (*YALI0E12463*) and *ylXKS* (*YALI0F10923*) were overexpressed in the PO1f strain under the control of a UAS1B8-TEF_min_ promoter and a *S. cerevisiae* CYC1 terminator, leading to growth on xylose up to approximately 3 g/L DCW, without the need for any adaptation (Rodriguez et al., 2016). This study went further to identify 14 homologs of the *ssXYR*, of which one enzyme, *ylXYR2 (YALI0F18590)*, had functional xylose reductase in an *Escherichia coli* complementation assay; however, its overexpression along with *ylXDH* and *ylXKS* did not result in any improvement in growth rate. Overexpression of the native *ylXDH*, *ylXKS*, and *ylXYR1* (*YALI0D07634*) under the strong constitutive TEF promoter resulted in very fast growth rates (Niehus et al., 2018). Combined, these results strongly suggest that *Y. lipolytica* has a cryptic metabolism of xylose that is difficult to activate unless under stressed conditions. These data also suggest that *Y. lipolytica*’s enzymes have high-specific activity and that its metabolism is already well primed for xylose utilization, unlike *S. cerevisiae*.

Two groups used heterologous genes to engineer xylose metabolism in *Y. lipolytica*. Li and Alper (2016) overexpressed *ssXYR* and *ssXDH* under a strong hybrid TEF promoter in both PO1f and an evolved lipid-producing strain, requiring a 14-day adaptation to find clones that grew well on xylose. Interestingly, they noted their adaptation resulted from genome duplication events during carbon starvation. Ledesma-Amaro et al. approached engineering xylose metabolism by recognizing that heterologous expression of *ssXYR* and *ssXDH* genes may be insufficient for growth of *Y. lipolytica* as they noted that xylose pathway engineering in *S. cerevisiae* required overexpression of an XKS to convert xylulose into xylulose-5-phosphate and for growth on xylose (Kotter et al., 1990). Using a BLAST search, a putative endogenous *ylXKS* (*YALI0F10923*) was found in *Y. lipolytica* (Ledesma-Amaro and Nicaud, 2016b; Rodriguez et al., 2016). Overexpression of the *ssXYR*, *ssXDK*, and *ylXKS* under the TEF promoter in the PO1d strain of *Y. lipolytica* resulted in growth on xylose of approximately 13 g/L, similar to wild-type growth on glucose, without the need for adaptation (Ledesma-Amaro et al., 2016b). An alternative approach to enable xylose metabolism in *Y. lipolytica* involved using a xylose isomerase (xylA) from *Piromyces* sp., which converts D-xylose into D-xylulose (**Figure 2**). By this process, the xylA effectively replaces the function of two enzymes, XYR and XDH, in the xylose pathway. Studies using strains overexpressing *xylA* instead of *XYR* and *XDH* together have resulted in strains unable to metabolize xylose, even with adaptive evolution (Li and Alper, 2016). Additionally, BLAST analysis of the xylA protein against *Y. lipolytica* revealed no homologs. Nonetheless, a pathway using xylA and XKS is possible and may be feasible with organism-specific codon-optimization, confirmation that the mRNA expression is optimal, and verification that the protein is being functionally expressed.

### L-Arabinose

Another pentose which is present in hemicellulose and could serve as an alternative carbon source is arabinose. Arabinose catabolism has not been well-characterized in *Y. lipolytica*; however, limited work has been done to elucidate this pathway (Ryu and Trinh, 2017). L-Arabinose metabolism has been engineered in the conventional yeast, *S. cerevisiae*, by pairing its endogenous galactose permease (*GAL2*) with bacterial arabinose metabolizing genes: L-Arabinose isomerase (*araA*) from *Bacillus subtilis*, L-ribulokinase (*araB*) from *E. coli*, and L-ribulose-5-phosphate 4-epimerase (*araD*) also from *E. coli* (Becker and Boles, 2003).

Protein BLAST using the *S. cerevisiae* GAL2 sequence as a query returned 47% identity with the endogenous *Y. lipolytica* protein, YALI0F19184p (**Figure 2**). Neither the *B. subtilis* araA nor the *E. coli* araD sequences returned any homologs in *Y. lipolytica*. One potential araB homolog, YALI0C07722p, was found to share 24% identity. This suggests that while genetic engineering may be necessary to completely enable L-arabinose metabolism in *Y. lipolytica*, the pathway for L-arabinose metabolism is possibly cryptic like the xylose pathway.

A second route for L-arabinose metabolism has been described for fungi (Chiang and Knight, 1960; Witteveen et al., 1989; Richard et al., 2003; Fonseca et al., 2007). In this pathway, L-arabinose is transported across the cell membrane with the same galactose permease, GAL2, described in the first pathway. It is then converted into L-arabinitol by an L-arabinose reductase. L-Arabinitol is subsequently turned into L-xylulose by an L-arabinitol 4-dehydrogenase. Finally, L-xylulose is converted into xylitol by an L-xylulose reductase, where it can be shuttled through the xylose interconversion pathway and eventually through the pentose phosphate pathway (**Figure 2**).

A fungal L-arabinose reductase (*larA*) from *Aspergillus niger* has been used to convert L-arabinose into L-arabinitol (Mojzita et al., 2010). To determine whether *Y. lipolytica* might contain a potential larA homolog for the first step in the breakdown of L-arabinose, BLASTp was used with larA as the query. Thirteen hits were found in this search, with the top hit, YALI0F18590p, sharing 93% coverage and 48% identity with the reference protein. Interestingly, the next five best hits all shared 90% coverage and 44% identity with larA. These results suggest that an L-arabinose metabolizing pathway is very likely to be present in *Y. lipolytica* either in whole or in part. The fungal L-arabinose 4-dehydrogenase (ladA), also from *A. niger*, was subjected to BLASTp to find *Y. lipolytica* homologs. In this case, nine hits resulted, with the top two hits, YALI0E12463p and YALI0F09603p, both sharing 86% coverage and 40 and 24% identity, respectively, with the ladA protein sequence. YALI0E12463p has been described in work with D-xylose utilization as a xylitol dehydrogenase (Ledesma-Amaro and Nicaud, 2016b; Li and Alper, 2016; Rodriguez et al., 2016). It is possible that overexpression of this protein in *Y. lipolytica* could enable both the conversion of L-arabinitol to L-xylulose and xylitol to D-xylulose. Because there were substantially more potential homologs found in *Y. lipolytica* using the fungal L-arabinose metabolizing pathway as a reference as opposed to the bacterial pathway, future studies into L-arabinose metabolism will likely focus on this fungal pathway.

The ALX1 protein from the L-arabinose metabolizing yeast, *Ambrosiozyma monospora*, was first identified as an L-xylulose reductase, which converts L-xylulose into xylitol (Verho et al., 2004). ALX1 has been successfully expressed in *S. cerevisiae* and, with the expression of an L-arabitol 4-dehydrogenase, growth was observed on media containing L-arabinose as the sole carbon source (Bera et al., 2010). Additionally, a protein BLAST was performed using the ALX1 protein to identify *Y. lipolytica* homologs. The top two hits from this search were YALI0F02211p, with 74% query coverage and 50% identity, and YALI0E05643p, with 98% query coverage and 38% identity (**Figure 2**). This suggests that ALX1 can be used as a pathway component to enable L-arabinose metabolism in yeast that do not metabolize L-arabinose naturally and that homologs of ALX1 may already be present in *Y. lipolytica*.

### Pentose Transporters

The mode by which pentose sugars are transported into *Y. lipolytica* has been debated. In strains that can metabolize D-xylose, for example, the transport mechanism is unclear. Co-utilization studies with glucose and xylose suggest that glucose transporters may be non-specifically transporting xylose across the yeast cell membrane (Sedlak and Ho, 2004). Using known xylose transporters as queries, 22 endogenous genes have been linked to xylose transport in *Y. lipolytica* through a BLAST analysis (Ryu et al., 2016b). Analysis of the mRNA expression showed that two of these genes, YALI0D01111 and YALI0C04730, were upregulated nearly 10-fold in xylose media (Ryu et al., 2016a). Thirteen other transporters were observed to be upregulated in xylose, suggesting that there could be multiple transporters which help to transport D-xylose across the cell membrane (Nicaud et al., 2017). More recently, Ryu and Trinh (2017) has reiterated that YALI0C04730 and YALI0B00396 function as pentose-specific transporters in *Y. lipolytica*. These data, along with the fact that xylose metabolism can be enabled with the overexpression of xylose pathway genes, suggest that xylose transport is not a bottleneck for cells utilizing D-xylose as a sole carbon source.

Alternatively, transporters from other yeasts have been engineered to be specific for sugars like D-xylose. A glucose/xylose transporter from *Candida intermedia*, for example, has been evolved to be a xylose-specific transporter in *S. cerevisiae* (Young et al., 2014). The main problem with this transporter is that the presence of glucose inhibits xylose transport in dual-substrate cultures. With the goal of enabling simultaneous glucose and xylose metabolism, the *ciGXS1* transporter was engineered by directed evolution, resulting in a transporter capable of co-utilization of glucose and xylose with no inhibition (Li et al., 2016). This approach may be useful in situations where transport of xylose across the cell membrane is a limiting factor, such as when higher concentrations of xylose or when multiple substrates are used.

### Outlook

The pentose metabolizing capabilities of *Y. lipolytica* have gained attention due to the abundance of pentose sugars in lignocellulosic biomass. With the ever-improving development of bioinformatics and metabolic engineering tools, endogenous sugar utilization pathways are emerging from the *Y. lipolytica* genome. Although arabinose metabolism has not been well-characterized in *Y. lipolytica*, the pathway for arabinose utilization may very well be present, yet cryptic, like xylose metabolism. There are still many questions to answer regarding xylose metabolism; however, progress has been made toward elucidating this pathway from both heterologous and endogenous overexpression of transporters and xylose metabolism enzymes in *Y. lipolytica*. Improvements which include co-utilization of more than two sugar substrates, more efficient utilization of sugars through cofactor use, and improving product yield will help to make the use of pentose sugars as a feedstock for *Y. lipolytica* oleochemical production industrially feasible. Another expected improvement is the use of xylan as a substrate for *Y. lipolytica* engineered for xylose metabolism.

## C2 and C3 Substrates

### Glycerol

Many reports characterize glycerol as the preferred substrate for *Y. lipolytica* based on its rapid growth rate (Workman et al., 2013). Furthermore, the tolerance of *Y. lipolytica* to inhibitory compounds found in crude glycerol justifies the great interest in engineering *Y. lipolytica* to convert unrefined glycerol byproduct of biodiesel production (Andre et al., 2009; Gao C.J. et al., 2016). As such, there is a large body of literature available, including several excellent reviews to which the reader is referred (Rywińska et al., 2013; Leiva-Candia et al., 2014; Samul et al., 2014; Vivek et al., 2017; Yan et al., 2017). For this reason, we do not consider glycerol or crude glycerol as an alternative substrate for the purposes of this review.

### Acetate

The use of acetate as a substrate for *Y. lipolytica* is promising due to its naturally high flux capacity for acetyl-CoA that is required for lipid efficient lipid accumulation. As a result, there is increasing interest in utilizing acetate or acetic acid as a substrate for *Y. lipolytica*. Furthermore, acetate can be produced from several low-cost sources, such as syngas (CO/H_2_), which is abundantly available from gasification of organic material, industrial off-gases, and anaerobic digestion using acetogens equipped with the Wood–Ljungdahl pathway (Lian et al., 2012; Bengelsdorf et al., 2013; Hu et al., 2013; Jiang et al., 2013). Growth on acetate requires its activation to acetyl-CoA in the cytoplasm, which is catalyzed by the acetyl-CoA synthetase (ACS) (Liu et al., 2016). Acetate metabolism was studied using ^13^C flux analysis, showing lipogenic NADPH is made through gluconeogenesis and the oxidative pentose phosphate pathway (Liu et al., 2015).

Recently, an engineered highly lipogenic strain of *Y. lipolytica* (Qiao et al., 2015) was grown in media with 30% v/v acetic acid to produce lipids with a titer of 51 g/L, a productivity 0.26 g/L-h, and 61% lipid content (Hu et al., 2016). In the same study, similar results (46 g/L, 0.27 g/L-h, 59% lipids) were achieved using a more dilute acetic acid solution (3% v/v) using a cell recycling strategy. This process was further optimized and converted to a semicontinuous process leading to 115 g/L, 0.16 g/g, and 0.8 g/L-h from a 3% dilute acetic acid media (Xu et al., 2017). Another strain of *Y. lipolytica* was engineered to produce 7.35 g/L polyhydroxybutyrate (PHB) (10.2% of cell dry weight) from a complex media with an acetate carbon source (Li et al., 2017).

### Outlook

Acetate is a promising low-cost substrate for growth and product formation in engineered *Y. lipolytica* strains. Its production from a variety of agroindustrial wastes make it attractive from a sustainability, and potentially economic, perspective. Furthermore, the abundance of acetate produced in biomass pretreatment could provide a significant low-cost source of acetate to use in bioprocesses (Yan et al., 2017). There has been significant progress in using acetate as an alternative substrate through bioprocess control strategies (Xu et al., 2017); however, metabolic engineering strategies have not been applied to improving the utilization of acetate. Such strategies include increased acetate tolerance, increased uptake, and improved co-utilization. It is also possible that combining acetate and sugars as a feedstock would provide an energetic advantage for certain bioprocesses.

## Lipids

### Triacylglycerides

Triacylglycerides are chemical species composed of three fatty acid chains attached to a glycerol backbone via ester bonds. TAGs serve as the primary form of excess carbon storage in oleaginous yeast, such as *Y. lipolytica*, and tend to form intracellular lipid bodies (LB) (Athenstaedt et al., 2006). In the presence of sufficient nitrogen and phosphorous, but upon carbon starvation, TAGs can be systematically catabolized to satisfy the carbon requirement. The initial degradation step is carried out by intracellular lipases.

**FIGURE 3:**
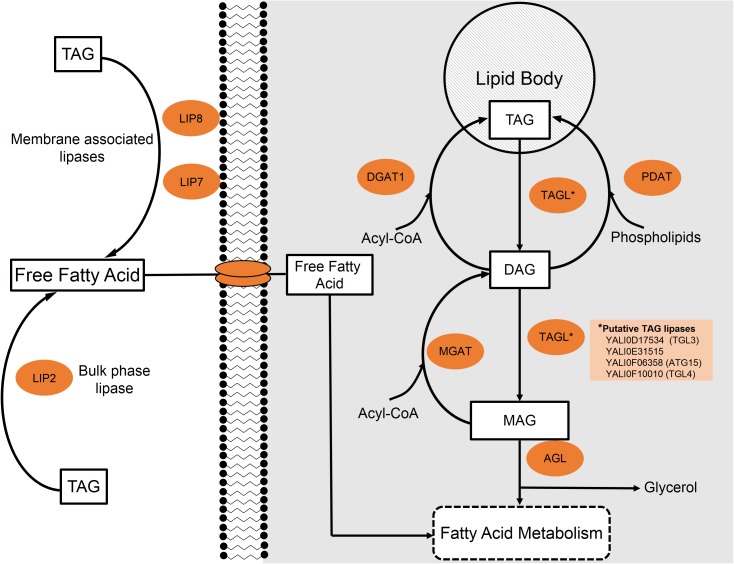
Lipid metabolism of triacylglycerides (TAGs) in *Y. lipolytica*. Extracellular and intracellular TAGs are processed through a stepwise degradation pathway focusing around either extracellular lipases (Lip2, Lip7, and Lip8) or internal triacylglycerol lipases (TGLs), respectively.

*Yarrowia lipolytica* possesses 16 genes encoding for lipases and one study suggests that 15 of the 16 lipases are capable of acting intracellularly (Syal and Gupta, 2017). Regardless of location, the first step involves the cleavage of an ester bond to form a free fatty acid and a diacylglycerol (DAG). **Figure 3** provides a general schematic of the degradation process. The same lipase can further hydrolyze DAG to produce a second free fatty acid and a monoacylglycerol (MAG). The final step to release a third fatty acid and glycerol is catalyzed by acylglycerol lipase (YALI0C14520p) (Zhao et al., 2011).

In order to metabolize extracellular TAGs as a carbon substrate, *Y. lipolytica* likely must first degrade TAG to free fatty acids in the extracellular environment as no known TAG transporters have been identified (Beopoulos et al., 2009). Three extracellular lipases have been characterized in the *Y. lipolytica* literature: LIP2 (YALI0A20350p), LIP7 (YALI0D19184p), and LIP8 (YALI0B09361p) (Fickers et al., 2005). In strains with all three of these lipase genes knocked out, cells were unable to grow on lipid substrates as the sole carbon source. Fickers et al. (2005) suggest that this is strong evidence supporting the claim that no other extracellular lipases exist in *Y. lipolytica*.

LIP2 makes up the majority of the expressed extracellular lipases and has been extensively studied (Pignede et al., 2000). Due to its high expression and secretion, LIP2 was used as one of the model systems for excretory enzymes in this yeast (Nicaud et al., 2002). Extracellular lipases LIP7 and LIP8, as characterized by Fickers et al. (2005), have amino acid sequence similarities to LIP2 of 33.9 and 35.2%, respectively. Compared to one another, LIP7 and LIP8 share 76% homology. The three extracellular lipases also possess differing substrate specificities. While all of these enzymes show a broad, normally distributed range of action, LIP2 tends to act on longer-chain lipids (Yu et al., 2007) and LIP7/LIP8 prefer shorter species, with the highest activity on 6-carbon/10-carbon compounds, respectively (Fickers et al., 2005).

The locations of these extracellular-acting lipases also differ. LIP2 is most commonly found in the bulk media during stationary phase; however, it is believed to be closely associated to the cell membrane during log phase growth. On the other hand, LIP7 and LIP8 appear to remain bound to cell membrane during the entire growth cycle; however, they may be released from the membrane under conditions of stationary phase carbon starvation (Fickers et al., 2005). Once the extracellular lipases have hydrolyzed TAGs, the free fatty acids and glycerol must be transported into the cell to be metabolized.

### Free Fatty Acids

**FIGURE 4:**
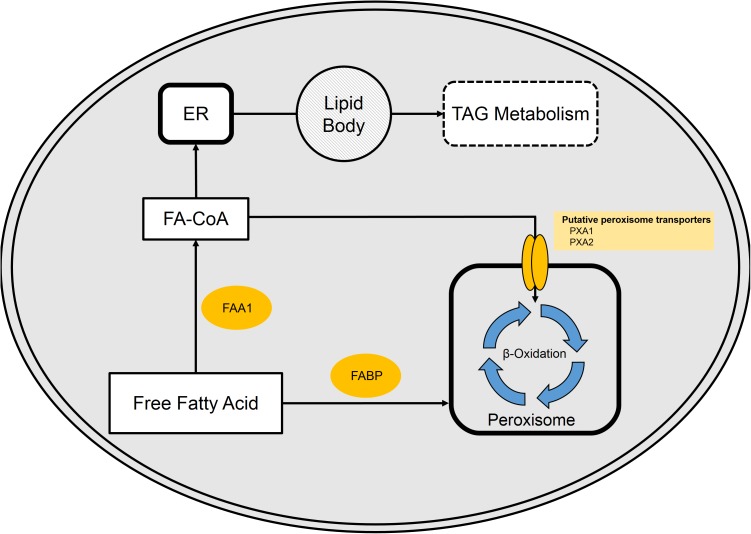
Free fatty acid (FFA) metabolism in *Y. lipolytica*. FFA present in the cytosol are bound by fatty acid-binding protein (FABP). Once bound, FAs either enter the peroxisome or are activated via fatty acyl-CoA synthase (FAA1). Activated FA (fatty acyl-CoAs) is either funneled into the peroxisome or enter the TAG synthesis pathway.

Uptake of free fatty acids from the bulk phase is believed to occur by either transporter or diffusion at concentrations above 10 μM (Kohlwein and Paltauf, 1984). Once in the cytoplasm, free fatty acids may be bound by a fatty acid-binding protein (FABP, YALI0E01298), activated by conversion into a fatty acyl-CoA by cytoplasmic fatty acyl-CoA synthase (FAA1, YALI0D17864p), or directly transported into the peroxisome (Dulermo et al., 2015; Ledesma-Amaro et al., 2016a). The fatty acyl-CoA enters the peroxisome via the membrane-bound transport protein pair PXA1 (YALI0A06655p) and PXA2 (YALI0D04246p) (Dulermo et al., 2015). The fatty acids/acyl-CoAs that enter the peroxisome can then be degraded by the well-studied β-oxidation cycle. Recent work from our group reveals synergistic effects of glycerol and fatty acid mixtures on the expression of β-oxidation gene, POX2 (Shabbir Hussain et al., 2017). Alternatively, the fatty acyl-CoA can enter the endoplasmic reticulum for incorporation into cellular lipids or TAGs (**Figure 4**).

### Olive Oil Mill Waste

Olive mill wastewater (OMW) is a waste stream produced by the processing of raw olives and contains water, olive tissue, sugars, polyphenolics, oils, and high amounts of salts – especially Na^+^, K^+^, and ^-^HCO_3_ species (Aly et al., 2014). One report estimates OMW production from Mediterranean countries alone at 30 million m^3^ – approximately 7.9 billion gallons (Mekki et al., 2013). OMW contains a substantial amount of phenolics that must be removed prior to further remediation and wastewater treatment (Aggelis et al., 2003).

*Yarrowia lipolytica*’s robust growth on a variety of substrates and tolerance for usually hostile growth media makes it an ideal candidate for remediation and valorization of OMW (Aly et al., 2014). Recent work demonstrated growth on glucose-supplemented OMW. A lipid composition of 48%, as well as 19 g/L citric acid production, was achieved on this mixed media (Sarris et al., 2017). Additionally, a post-fermentation assay of OMW phenolic concentration showed a decrease of up to 13.8%, suggesting a potential route for simultaneous remediation and valorization.

### Animal Fats

Much of the literature focuses on the production of lipids in *Y. lipolytica*; however, as the name suggests, it has a robust capability to break down fats and oils (lipolytic) as a carbon source. Of particular interest is waste animal fats. With relatively low market value, valorization of this waste stream has substantial economic potential. Kamzolova et al. (2005) characterized *Y. lipolytica*’s growth and lipase production on rendered beef fat. The authors demonstrated biomass accumulations of between 2.5 and 5.3 g/L DCW from media containing 10 g/L animal fat and 10 g/L peptone. Citric acid and isocitric acid yields of up to 18 and 5.2 g/L, respectively, were achieved on the animal fat containing media. Growth has also been demonstrated on derivatives of animal waste fat. Stearin (fat derivative), glucose, and waste glycerol were used to produce 3.4 g/L of a cocoa-butter-like lipid mixture high in stearic acid (Papanikolaou et al., 2003). In order to reach this yield, a high carbon-to-nitrogen ratio of 175:1 and the emulsifying agents Tween 80 and PEG 20,000 were required.

### Agro-Industrial Waste

Waste cooking oil (WCO) has already received substantial attention for its role as a synthetic biodiesel feedstock; however, it may also have potential as a substrate for *Y. lipolytica*. Demonstrating the flexibility of *Y. lipolytica* to convert a dissimilar feed to a new product, Xiaoyan et al. (2017) used WCO to produce the sugar alcohol erythritol. Currently, commercial erythritol is biologically produced from higher-cost glucose that has often been obtained via enzymatic hydrolysis of starch or other polysaccharides (Aoki et al., 1993). The cost of this multi-step, expensive procedure could potentially be greatly reduced by making use of non-sugar waste streams, such as WCO.

Unlike most of the aforementioned feedstocks, which serve primarily as sources of carbon, crustacean waste has been investigated as a potential nitrogen source to support the growth of *Y. lipolytica* (Magdouli et al., 2017). In their work, Magdouli et al. (2017) made use of crude glycerol and crustacean waste to produce 38 U/mL of lipase in *Y. lipolytica*. They also demonstrated similar levels of lipase production on other carbon waste sources such as used vegetable, olive oil, and motor oil.

The growing demand for palm oil has resulted in an increased supply of the waste product palm kernel cake (PKC). Of particular interest, this solid material contains 16.1% (w/w) protein and, when supplemented with glucose, was able to support the growth of *Y. lipolytica* (Imandi et al., 2010). Under these conditions, lipase yields of 18.58 U/g of PKC were possible.

### Outlook

The turn toward waste streams as potential feedstocks has opened up many potential routes for the symbiosis of biotechnology and sustainable manufacturing practices. Bioremediation, waste valorization, and *in situ* processing of byproducts present possible large-scale applications of appropriately engineered microorganisms. The myriad alternative carbon and nitrogen sources on which *Y. lipolytica* can grow suggests that it has the necessary properties to fill such a role. The advancement of bioinformatics tools allows for increasingly complex and accurate modeling of species fluxes within the organism. As the available genetic toolbox grows, important questions must be answered for further advances. Specifically, much is still unknown regarding the interaction between pathways, their activation, and their inhibition. This is of particular interest when considering growth on complex, undefined, and often inhospitable feeds. Metabolic engineering efforts to utilize waste substrates are likely to focus on relieving inhibitory effects of substrate contaminants and relieving cross-pathway regulatory effects.

## Conclusion

There is considerable interest in the use of alternative substrates due to bioprocess economics and sustainability. This interest is justified only if microbial systems can be engineered to efficiently use these substrates and tolerate contaminants and the regulatory effects of multiple species that can be metabolized. *Y. lipolytica* is well poised to enable the use of alternative feedstocks at large scale due to its natural tolerance to many chemical conditions and the wide array of products it can easily be engineered to produce. We expect future work in the field to begin applying metabolic engineering strategies to better utilize diverse and often unrefined substrates.

## Author Contributions

MSH, LG, MS, and MB participated in writing, editing, and approving the final manuscript.

## Conflict of Interest Statement

The authors declare that the research was conducted in the absence of any commercial or financial relationships that could be construed as a potential conflict of interest.
